# Definition of a Family of Tissue-Protective Cytokines Using Functional Cluster Analysis: A Proof-of-Concept Study

**DOI:** 10.3389/fimmu.2014.00115

**Published:** 2014-03-17

**Authors:** Manuela Mengozzi, Peter Ermilov, Alexander Annenkov, Pietro Ghezzi, Frances Pearl

**Affiliations:** ^1^Brighton and Sussex Medical School, Falmer, UK; ^2^Bone and Joint Research Unit, Bart’s and The London School of Medicine, William Harvey Research Institute, Queen Mary University of London, London, UK; ^3^Translational Drug Discovery Group, School of Life Sciences, University of Sussex, Falmer, UK

**Keywords:** cytokines, bioinformatics, cluster analysis, neuroprotection, tissue-protection, EGR2, early genes, repair

## Abstract

The discovery of the tissue-protective activities of erythropoietin (EPO) has underlined the importance of some cytokines in tissue-protection, repair, and remodeling. As such activities have been reported for other cytokines, we asked whether we could define a class of tissue-protective cytokines. We therefore explored a novel approach based on functional clustering. In this pilot study, we started by analyzing a small number of cytokines (30). We functionally classified the 30 cytokines according to their interactions by using the bioinformatics tool STRING (Search Tool for the Retrieval of Interacting Genes), followed by hierarchical cluster analysis. The results of this functional clustering were different from those obtained by clustering cytokines simply according to their sequence. We previously reported that the protective activity of EPO in a model of cerebral ischemia was paralleled by an upregulation of synaptic plasticity genes, particularly early growth response 2 (EGR2). To assess the predictivity of functional clustering, we tested some of the cytokines clustering close to EPO (interleukin-11, IL-11; kit ligand, KITLG; leukemia inhibitory factor, LIF; thrombopoietin, THPO) in an *in vitro* model of human neuronal cells for their ability to induce EGR2. Two of these, LIF and IL-11, induced EGR2 expression. Although these data would need to be extended to a larger number of cytokines and the biological validation should be done using more robust *in vivo* models, rather then just one cell line, this study shows the feasibility of this approach. This type of functional cluster analysis could be extended to other fields of cytokine research and help design biological experiments.

## Introduction

The cytokine field was initially focused on the role of cytokines as humoral factors in host defense against infection and cancer, and the discoveries of interferon (IFN), interleukins (IL)-1 and -2 (1). This led to exploring their use in the therapy of viral infections and cancer, and IFN is now used for the therapy of some viral diseases. However, the big bang in the field took place with the discovery of the proinflammatory activity of some cytokines, particularly IL-1 and TNF (2, 3), as this concept led to the development of new anti-inflammatory agents such as anti-TNF antibodies and soluble receptors, used in millions of patients.

The current focus on stem cell therapy and regenerative medicine has put an emphasis on the protective functions of some cytokines. Our earlier finding that erythropoietin (EPO) has neuroprotective activities in models of brain and spinal cord injury (4) led to the subsequent discovery of protective actions of EPO outside the CNS and to the use of the term tissue-protective cytokine [reviewed in Ref. (5, 6)]. Although this term had since been used for other cytokines (7), it has recently been used to define EPO or EPO-derived molecules, such as carbamylated EPO or EPO-derived peptides (8, 9).

It should be noted, however, that protective/regenerative functions of some cytokines have long been known. For instance transforming growth factor beta (TGFb) has been extensively studied in the context of wound repair (10), and IL-6 for increasing myelination (11).

We recently performed a microarray study to investigate the effect of EPO on the gene expression profile in rat ischemic brain and identified several genes associated with neuronal plasticity (12). These included early growth response 2 (EGR2), which is a transcription factor important in myelination. In fact, we could show that EPO promotes myelination, thus suggesting some similarity with IL-6 (13).

Another major action of some cytokines that can be regarded as protective/reparative is the stem cells-mobilizing action of EPO, Kit ligand/stem cell factor (KITLG), G-CSF, and GM-CSF (14, 15).

Despite studies in the literature reporting protective or reparative actions of various cytokines, no attempt has been made to define a functional family of tissue protective cytokines [see Ref. (16) for a review].

In the present study, we identify a cluster of tissue-protective cytokines using a bioinformatics approach. In particular, we have used the STRING (Search Tool for the Retrieval of Interacting Genes/Proteins) database (17). The STRING database integrates data from multiple experimental repositories that describe protein–protein physical and functional associations. These data are then augmented by predicted functional associations, from a number of complementary algorithms that utilize evolutionary information. STRING also identifies putative protein–protein associations using a text-mining algorithm. We used STRING to identify a comprehensive set of interactors for EPO and 29 other cytokines that belong to different structural families. We then identified the subset of these interactors that were related to tissue-protection or regeneration functions using the gene ontology (GO) classification performed by DAVID (Database for annotation, visualization, and integrated discovery) (18). The cytokines were grouped into functional subclasses based on their tissue-protective interactors using standard hierarchical cluster analysis. We predicted that those cytokines that cluster close to EPO would be similar in terms of tissue-protective functions.

To assess the effectiveness of this strategy, four cytokines were chosen and their ability to protect tissue from ischemic damage was assessed experimentally by determining the degree to which they induce EGR2 expression in neuronal cells, an experimental model in which EPO shows activity (12).

We find that this strategy is far more effective at identifying cytokines with common biological functions than clustering simply based on amino acid sequence similarity.

## Materials and Methods

### Identification of cytokine interactors using STRING

A list of functional interactors for each cytokine was obtained using the STRING database, available online^1^ (17). Thirty cytokines, including EPO (Table 1) were input into STRING to obtain a list of functional interactors. STRING assigns to each reported functional association a confidence score, which is dependent on both the experimental method on which the functional association prediction is based, and on the reliability of computational approaches used for prediction, so that each functional association can be confidence weighted as a measure of reliability.

**Table 1:** **List of cytokines studied in the cluster analysis**.

Cytokine	Abbreviation
Brain-derived neurotrophic factor	BDNF
Cardiotrophin-like cytokine factor 1	CLCF1
Ciliary neurotrophic factor	CNTF
Cardiotrophin 1	CTF1
Erythropoietin	EPO
Granulocyte colony stimulating factor	G-CSF
Growth hormone	GH
Granulocyte-macrophage colony stimulating factor (CSF2)	GM-CSF
Interferon alpha 1	IFNA-1
Interleukin-10	IL-10
Interleukin-11	IL-11
Interleukin-12A	IL-12A
Interleukin-18	IL-18
Interleukin-1 alpha	IL-1A
Interleukin-1 beta	IL-1B
Interleukin-2	IL-2
Interleukin-21	IL-21
Interleukin-3	IL-3
Interleukin-4	IL-4
Interleukin-5	IL-5
Interleukin-6	IL-6
Interleukin-1 receptor antagonist	IL-1RN
Kit ligand/stem cell factor	KITLG
Leptin	LEP
Leukemia inhibitory factor	LIF
Nerve growth factor	NGF
Oncostatin M	OSM
Prolactin	PRL
Thrombopoietin	THPO
Tumor necrosis factor alpha	TNF

For each cytokine, search criteria were: a maximum of 500 interactors, combined confidence score >0.2 and use of all active prediction methods, including text mining. No “white nodes” were requested in the search criteria. This refers to secondary interactors, proteins that are predicted functional partners of primary interactors with the search cytokine, but which do not interact directly with the search cytokine.

Data for each cytokine was saved as a text file and imported to Microsoft Excel for processing. Primary interactors with each cytokine and the combined confidence score for each respective interaction were extracted from all 30 datasets and pooled onto a single spreadsheet. The lowest scoring interactions that straddled the 500 interactors search limit were deleted as it would not be clear how many other predicted functional interactors with this score existed without increasing the upper search limit past 500. A Perl program was used to integrate all the interaction data into a single data matrix for further analysis.

### Functional annotation

The interactors were functionally annotated using the DAVID database, accessible online^2^ (18).

### Cluster analyses

Clustalw^3^ was used to cluster the proteins by sequence similarity, whereas the functional interactors patterns were analyzed using the Genesis software^4^ (Version 1.7.6 for Mac OSX).

### Cell culture

The rat neuroblastoma B104 cell line, genetically modified to express EPOR constitutively, as reported (12), was cultured in DMEM high-glucose (PAA Laboratories, Yevil, UK) supplemented with 10% (vol/vol) FBS, 100 U/ml penicillin and 100 μg/ml streptomycin (Invitrogen/Life Technologies, Carlsbad, CA, USA; complete medium). Before cytokine treatment, cells were switched to medium without serum with 5 μg/ml insulin, 5 μg/ml transferrin, and 5 ng/ml selenium (Sigma-Aldrich, St. Louis, MO, USA) and incubated for 4 h. Cells were treated with 80 ng/ml recombinant human EPO (rhEPO; Creative Dynamics, New York, NY, USA), 20 ng/ml recombinant mouse leukemia inhibitory factor (rmLIF; Sigma-Aldrich), 50 ng/ml recombinant human interleukin-11 (rhIL-11), 50 ng/ml recombinant rat KITLG (rrKITLG), or 50 ng/ml recombinant human thrombopoietin (rhTHPO) (all from R&D Systems, Minneapolis, MN, USA). After 1 h, total RNA was extracted and Egr2 mRNA quantified by qPCR.

### qPCR

Total RNA was extracted from cultured cells using TRIzol (Invitrogen/Life Technologies). RNA quality and concentration were determined using a NanoDrop ND-1000 (NanoDrop Technologies/Thermo Fisher Scientific, Wilmington, DE, USA). Reverse transcription and real time qPCR were carried out as reported (12), using TaqMan gene expression assays for rat Egr2 and rat glyceraldehyde 3-phosphate dehydrogenase (GAPDH, housekeeping gene), commercially available from Applied Biosystems/Life Technologies). For quantification, we used the comparative threshold cycle (ΔΔCt) method, following Applied Biosystems/Life Technologies guidelines. Results were normalized to GAPDH and expressed as arbitrary units, using as a calibrator one of the control samples. Statistical significance was determined using the unpaired two-tailed Student *t*-test.

## Results

**Figure 1:**
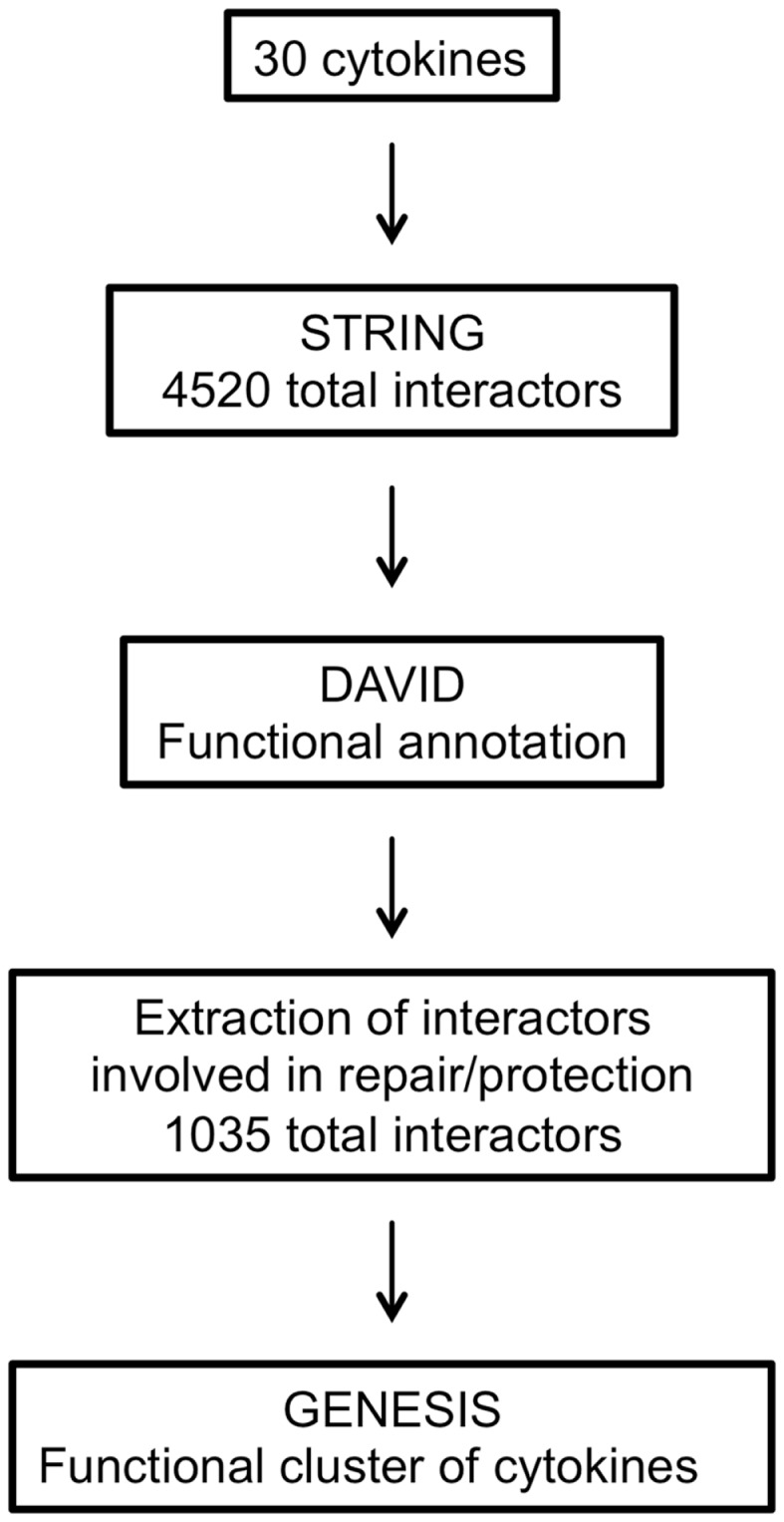
**Flow chart summarizing the steps involved in data processing**.

The main steps involved in data processing are summarized in Figure 1. The primary interactors for 30 cytokines including EPO (Table 1) were identified using the STRING database and the combined confidence score for each respective interaction extracted. In total, 4250 proteins were identified as having a functional association with at least one of the proteins in the data set.

To compare the patterns of interaction of the cytokines, a matrix was constructed that combined all the interactions identified. The matrix was populated by the interaction confidence for each cytokine, scored against all 4250 identified interactors. Where a functional interaction had been identified by STRING, the interaction was defined by the combined confidence score. If no interaction was identified, a score of −1 was recorded.

### Extraction of interactors involved in tissue-protection using DAVID

All the interactors were annotated using the DAVID functional annotation resource (see text footnote 2) and GO terms were assigned to each. Interactors with no associated GO terms were excluded, thus obtaining a dataset of 4338 interactors out of 4520.

Since our aim was to identify cytokines functionally similar in terms of protective/reparative functions, therefore sharing interactors involved in “repair or protection,” we chose to select only the interactors identified by GO terms containing the text listed in Table 2.

**Table 2:** **List of GO terms text strings used**.

Plasticity
Wound healing
Remodeling
Regeneration
Apoptosis
Angiogenesis
Neurogenesis
Neuron differentiation
Myelination

The respective interactors were then extracted from the complete list. Therefore, only 1035 interactors involved in repair or protection were used for the subsequent cluster analysis (Table S1 in Supplementary Material).

### Cluster analysis

**Figure 2:**
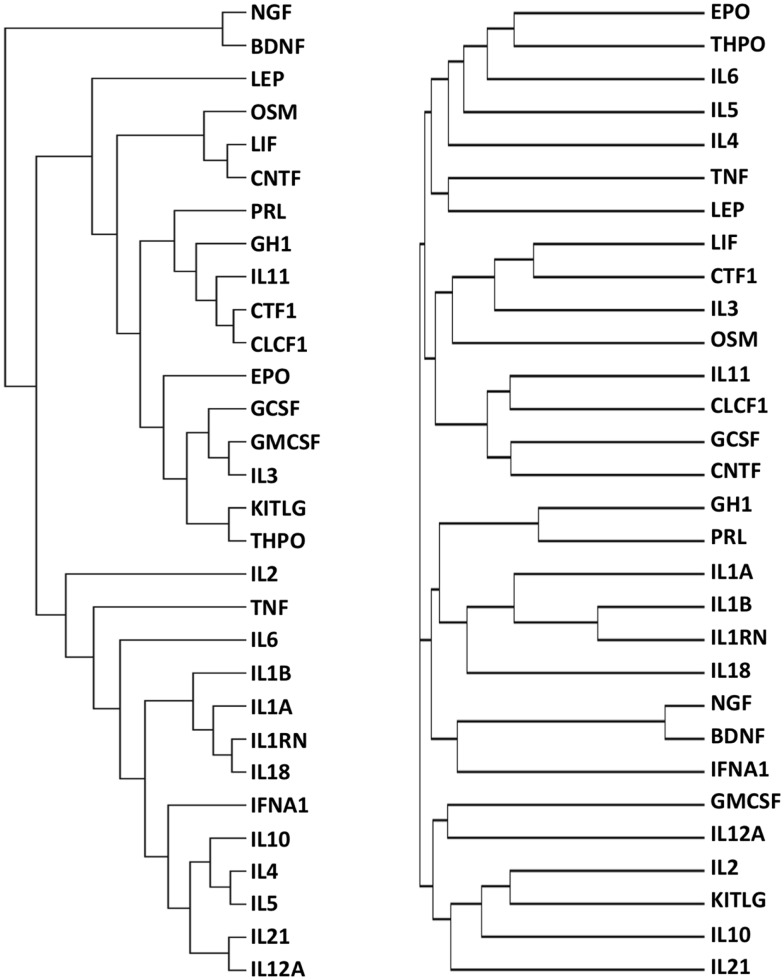
**Functional (left) and sequence (right) clustering of 30 cytokines**. Cytokines were functionally clustered, using Genesis, according to shared interactors involved in “repair/protection”, identified by GO terms containing the text listed in Table 2 (left) or according to their sequence, using ClustalW2 (right).

The final dataset, consisting of the 30 cytokines scored against the 1035 genes extracted with DAVID (Table S1 in Supplementary Material), was imported into the GENESIS analysis suite (Genesis version 1.7.6 for Mac OSX). Hierarchical clustering was then performed and the results are shown in Figure 2, left panel. For purpose of comparison, Figure 2, right panel, shows the results of clustering proteins according to their sequence using ClustalW2 (see text footnote 3), visualized as distance trees with JalView^5^.

It can be seen that the distribution of cytokines within primary, secondary, and tertiary clusters to EPO are markedly different in the “functional” cluster and in the sequence alignment cluster.

### Using functional cluster analysis to predict biological activity

Cytokines selected from primary, secondary, and tertiary clusters with respect to EPO (IL-11, KITLG, LIF, THPO) were chosen as experimental candidates, to test their effect on Egr2 mRNA expression in serum-starved EPOR-B104 neuroblastoma cells exactly as we described previously (12).

**Figure 3:**
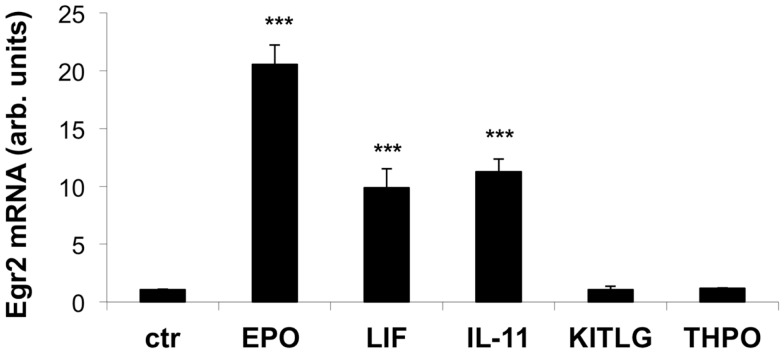
**Effect of EPO, LIF, IL-11, KITLG, and THPO on Egr2 mRNA in neuronal cells**. Cells were plated in 24-well plates at 120,000 cells/ml in complete medium. After overnight incubation, the cells were deprived of serum for 4 h and then stimulated with EPO (80 ng/ml), LIF (20 ng/ml), IL-11 (50 ng/ml), KITLG (50 ng/ml), or THPO (50 ng/ml) for 1 h. Egr2 mRNA was measured by qPCR, using GAPDH as a housekeeping gene. Results represent the change in expression level vs. one of the control (ctr) samples, and are the mean ± SD of triplicate samples assayed in duplicate. One representative experiment out of three is shown. ***P < 0.001 by Student’s*t*-test.

As shown Figure 3, EPO, IL-11, and LIF significantly increased the expression of Egr2 mRNA after serum deprivation when compared to a control sample. Neither KITLG nor THPO at 50 ng/ml elicited a significant elevation in Egr2 mRNA expression, at least in the experimental conditions used.

### Identification of genes involved in tissue-protection

**Figure 4:**
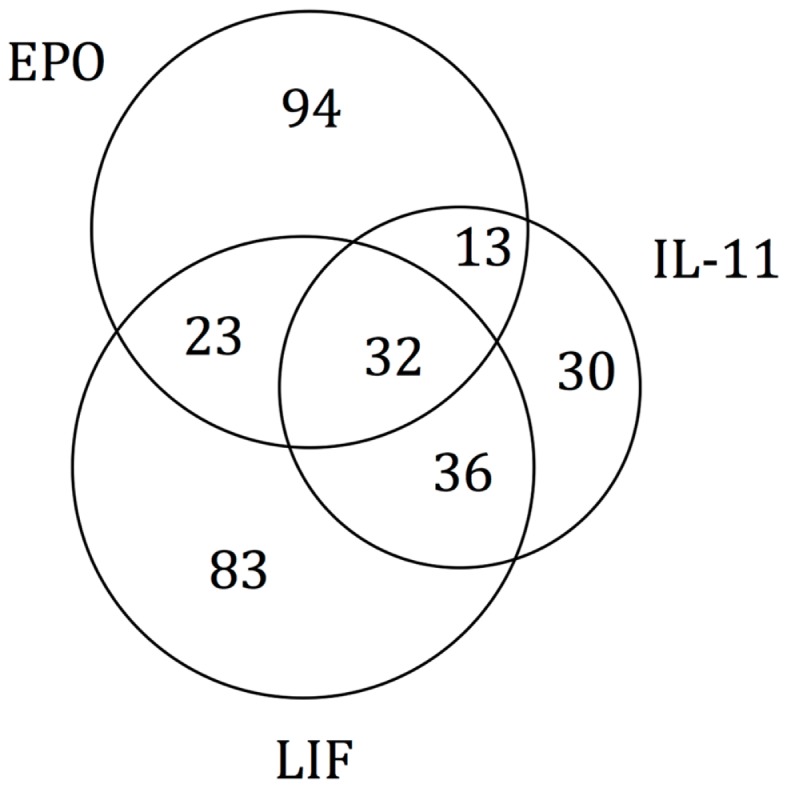
**Venn diagram of EPO, LIF, and IL-11 interactors**. A Venn diagram was constructed to identify commonalities in the interaction partners of EPO, LIF, and IL-11. Total number of interactors for each cytokine: EPO, 162; IL-11, 111; LIF, 174. The 32 interactors common to EPO, IL-11, and LIF are listed in Table 3.

As the experimental results confirmed that EPO, LIF, and IL-11 exhibit similar tissue protective functions, we decided to explore the similarities on their protein interaction networks. We identified the genes predicted to have a functional association with EPO, LIF, and IL-11. Venn diagrams were used to display the similarities among EPO, LIF, and IL-11 in terms of shared interactors (Figure 4). Thirty-two genes were predicted to have functional associations with all three cytokines (Table 3).

**Table 3:** **List of interactors common to EPO, LIF, and IL-11**.

Gene symbol	Gene name
BCL2	B-cell CLL/lymphoma 2
CSF2	Colony stimulating factor 2 (GM-CSF)
EFNB1	Ephrin B1
EP300	E1A binding protein p300
FOSL1	FOS-like antigen 1
IGF1	Insulin-like growth factor 1
IL10	Interleukin 10
IL12B	Interleukin 12B
IL1A	Interleukin 1 alpha
IL1B	Interleukin 1 beta
IL2	Interleukin 2
IL3	Interleukin 3
IL6	Interleukin 6
IL6R	Interleukin 6 receptor
IL7	Interleukin 7
INS	Insulin
JAK2	Janus kinase 2
JUN	Jun oncogene
LIFR	Leukemia inhibitory factor receptor alpha
OSM	Oncostatin M
PTH	Parathyroid hormone
PTPN6	Protein tyrosine phosphatase, non-receptor type 6
PTPRC	Protein tyrosine phosphatase, receptor type, C
SMAD1	SMAD family member 1
SOCS3	Suppressor of cytokine signaling 3
SOD1	Superoxide dismutase 1
SOD2	Superoxide dismutase 2
STAT1	Signal transducer and activator of transcription 1
STAT3	Signal transducer and activator of transcription 3
STAT5A	Signal transducer and activator of transcription 5A
TGFB1	Transforming growth factor beta 1
TNF	Tumor necrosis factor

**Figure 5:**
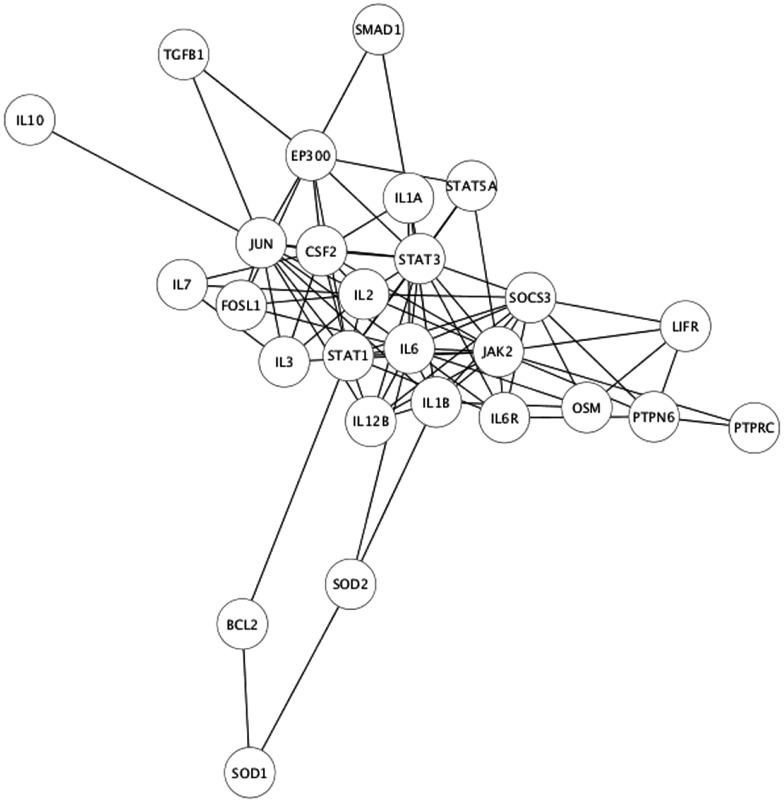
**Interaction network of the 27 connected, common interactors**.

*The list of the 32 interactors common to EPO, LIF, and IL-11 was input to STRING (confidence score >0.2, no text mining) and visualized as a network. Only five nodes (EFNB1, IGF1, INS, PTH, and TNF) were isolated and were therefore excluded. The remaining 27 common interactors were input to Cytoscape to construct a biological association network (Figure 5)*.

### Identification of other functional families

**Figure 6:**
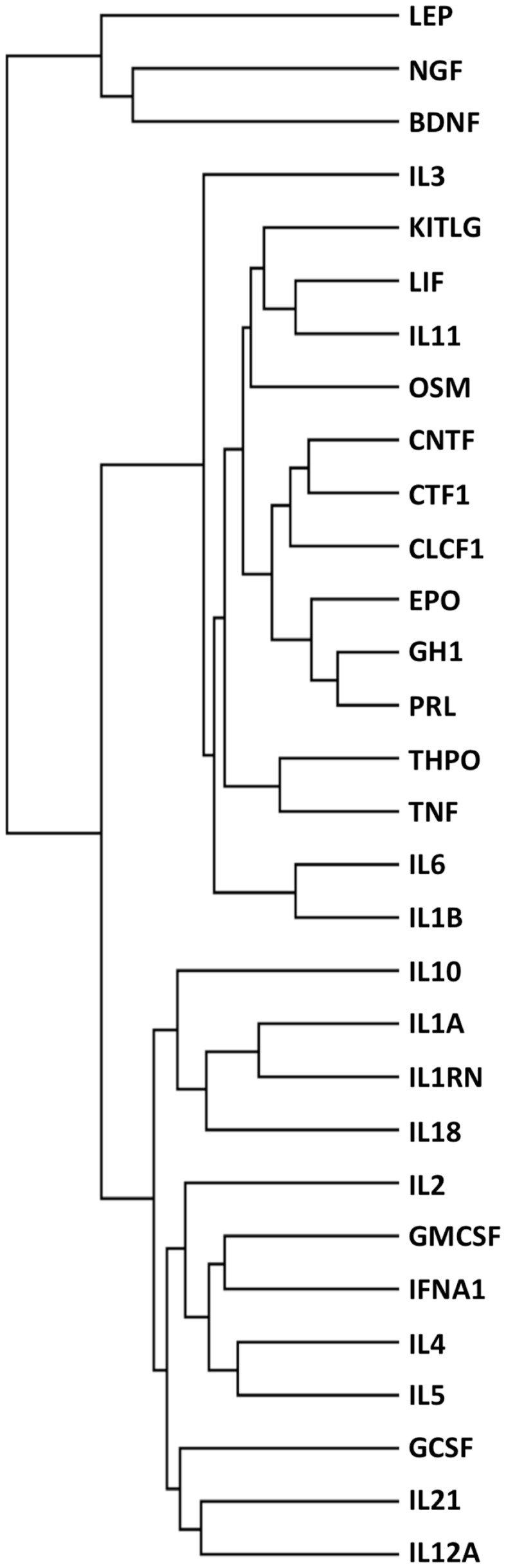
**Functional clustering of 30 cytokines for their role in inflammation**. Cytokines were functionally clustered, using Genesis, according to shared interactors involved in inflammation, as identified by their GO terms.

To further explore the potential of this strategy, we also performed an analysis where cytokines were functionally clustered in terms of inflammatory functions. To this aim, we selected only the interactors having “inflammatory” in their GO terms, and obtained 299 interactors (Table S2 in Supplementary Material). We then clustered the cytokines based on these interactors (Figure 6), and obtained functional families that were clearly different from those obtained when clustering according to “tissue-protective” interactors (Figure 2, left panel). In particular, when cytokines were clustered according to their “inflammation-related” interactors (Figure 6), EPO was closest to GH1, PRL, and THPO, whereas in terms of tissue-protective functions EPO was closest to G-CSF, GM-CSF, and IL-3 (Figure 2, left panel), known to share tissue-protective activities with EPO using in certain models the same signaling pathway through the common beta chain receptor (6). Therefore, this strategy gives different results based on the different interactors that are selected and used for the cluster analysis.

## Discussion

This study shows a pragmatic approach to identify a functional class of cytokines. In particular, we have tested the possibility of defining a functional family of neuro- and tissue-protective cytokines. We have also validated the results of the prediction by testing some of the cytokines *in vitro*.

While, ideally, the way to validate the predictions would have been to test all 30 cytokines in multiple *in vivo* models (e.g., myocardial infarction, cerebral ischemia, wound healing) this was neither feasible nor within the scopes of this study and we decided to use an *in vitro* test for this pilot study. In fact we could demonstrate that IL-11 and LIF can induce EGR2 expression in a system where EPO is effective. Of note, the induction of EGR2 expression by LIF or IL-11, in neurons as well as in other cells, was not previously known, indicating that functional clustering can actually be helpful in predicting an unknown biological activity.

However, one limitation of this study is that the *in vitro* experiments are not necessarily confirmative because the cell line used may not express sufficient levels of the receptor for the specific cytokine, and this could be the reason for the lack of activity of KITLG and THPO in our system. Another limitation is that the *in vitro* model used in this study (a human neuroblastoma cell line) is obviously tailored toward the nervous system and is not appropriate for studying other tissue-protective activities. For instance, KITLG administration via a lentiviral vector was reported to protect against myocardial infarctions, and the assay we used is clearly unsuitable to study a cardioprotective activity. Furthermore, *in vivo* testing using the appropriate models depending on the functional family explored would be desirable to validate the results obtained with the cluster analysis.

The observed induction of EGR2 by LIF and IL-11 might be important in the common pro-myelinating effects of these cytokines (19, 20), as EGR2, also known as KROX-20, is a transcription factor involved in myelination (21).

The identification of common interactors for EPO, LIF, and IL-11 (listed in Table 3) might also shed light on the mechanism of actions of tissue-protective cytokines. These common genes include a number of other cytokines as well as signaling molecules belonging to the JAK/STAT pathways that were easily predictable. However, they also include JUN and FOSL1, that are among the early genes induced by growth factors in the nervous system (22), genes involved in apoptosis pathway, such as BCL2 (23), and neuroprotective factors such as IGF1 (24), that is also involved in myelination (25).

In conclusion, this study reports for the first time a strategy to cluster cytokines by their function. The results, although performed on a limited number of cytokines, support the concept of a functional family of tissue-protective cytokines that share the tissue-protective activities of EPO.

Finally, although this study was aimed at defining a class or “tissue-protective cytokines,” we also tried to use the same strategy to identify other functional families by choosing to cluster only for interactors containing other specific GO terms, in particular only those related to inflammation. Indeed, EPO clustered with different cytokines depending on the interactors selected for the cluster analysis.

We therefore propose that this approach, if performed on a large number of cytokines, will be helpful in identifying other functional families.

## Conflict of Interest Statement

The authors declare that the research was conducted in the absence of any commercial or financial relationships that could be construed as a potential conflict of interest.

## Supplementary Material

The Supplementary Material for this article can be found online at: www.frontiersin.org/journal/10.3389/fimmu.2014.00115/abstract

## Footnotes

^1^http://string-db.org/

^2^http://david.abcc.ncifcrf.gov/

^3^http://www.ebi.ac.uk/Tools/msa/clustalw2/

^4^https://genome.tugraz.at/genesisclient/genesisclient_description.shtml

^5^http://www.jalview.org/
